# *catena*-Poly[[dichloridomercury(II)]-μ-1,4-bis­[(pyridin-2-yl)meth­oxy]benzene-κ^2^ *N*:*N*′]

**DOI:** 10.1107/S1600536811038323

**Published:** 2011-09-30

**Authors:** Ying Liu, Hong-Sen Zhang, Ming-Xing Hu, Guang-Feng Hou, Jin-Sheng Gao

**Affiliations:** a Department of Materials and Chemical Engineering, Heilongjiang Institute of Technology, Harbin 150050, People’s Republic of China; b Modern Analysis, Test and Research Center, Heilongjiang Institute of Science and Technology, Harbin 150027, People’s Republic of China; c College of Chemistry and Materials Science, Heilongjiang University, Harbin 150080, People’s Republic of China

## Abstract

In the title compound, [HgCl_2_(C_18_H_16_N_2_O_2_)]_*n*_, the Hg^II^ atom is four-coordinated in a distorted tetra­hedral environment defined by two Cl atoms and two N atoms from two 1,4-bis­(pyridin-2-ylmeth­oxy)benzene ligands. The ligand shows a non-coplanar conformation, in which the dihedral angles between the two terminal pyridine rings and the linking benzene ring are 7.275 (17) and 74.020 (14)°. The flexible ligands link the Hg^II^ atoms into a chain running along [010], with an Hg⋯Hg separation of 10.335 (5) Å, which is equal to the *b* axis. The chains are connected by C—H⋯O and C—H⋯Cl hydrogen bonds.

## Related literature

For the synthesis of the ligand and general background to flexible pyridyl-based ligands, see: Liu *et al.* (2010*a* ▶,*b* ▶); Wang *et al.* (2007 ▶).
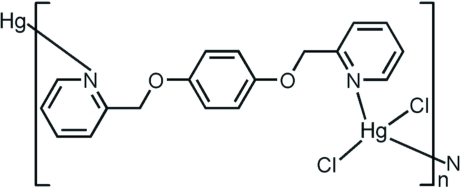


## Experimental

### 

#### Crystal data

[HgCl_2_(C_18_H_16_N_2_O_2_)]
*M* *_r_* = 563.82
Triclinic, 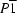

*a* = 9.201 (5) Å
*b* = 10.335 (5) Å
*c* = 11.040 (6) Å
α = 86.11 (2)°
β = 66.51 (2)°
γ = 73.860 (18)°
*V* = 923.8 (9) Å^3^
*Z* = 2
Mo *K*α radiation
μ = 8.63 mm^−1^
*T* = 293 K
0.19 × 0.17 × 0.17 mm

#### Data collection

Rigaku R-AXIS RAPID diffractometer
Absorption correction: multi-scan (*ABSCOR*; Higashi, 1995 ▶) *T*_min_ = 0.288, *T*_max_ = 0.325
9080 measured reflections
4179 independent reflections
3688 reflections with *I* > 2σ(*I*)
*R*_int_ = 0.051

#### Refinement

*R*[*F* ^2^ > 2σ(*F* ^2^)] = 0.036
*wR*(*F* ^2^) = 0.122
*S* = 1.16
4179 reflections
226 parameters
H-atom parameters constrained
Δρ_max_ = 1.56 e Å^−3^
Δρ_min_ = −1.74 e Å^−3^

### 

Data collection: *RAPID-AUTO* (Rigaku, 1998 ▶); cell refinement: *RAPID-AUTO*; data reduction: *CrystalStructure* (Rigaku/MSC, 2002 ▶); program(s) used to solve structure: *SHELXS97* (Sheldrick, 2008 ▶); program(s) used to refine structure: *SHELXL97* (Sheldrick, 2008 ▶); molecular graphics: *DIAMOND* (Brandenburg, 1999 ▶); software used to prepare material for publication: *SHELXTL* (Sheldrick, 2008 ▶).

## Supplementary Material

Crystal structure: contains datablock(s) I, global. DOI: 10.1107/S1600536811038323/hy2469sup1.cif

Structure factors: contains datablock(s) I. DOI: 10.1107/S1600536811038323/hy2469Isup2.hkl

Additional supplementary materials:  crystallographic information; 3D view; checkCIF report

## Figures and Tables

**Table 1:** Hydrogen-bond geometry (Å, °)

*D*—H⋯*A*	*D*—H	H⋯*A*	*D*⋯*A*	*D*—H⋯*A*
C2—H2⋯Cl1^i^	0.93	2.82	3.692 (8)	157
C11—H11⋯O2^ii^	0.93	2.51	3.338 (9)	149

Symmetry codes: (i) 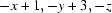; (ii) 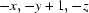.

**Fig. 1.**
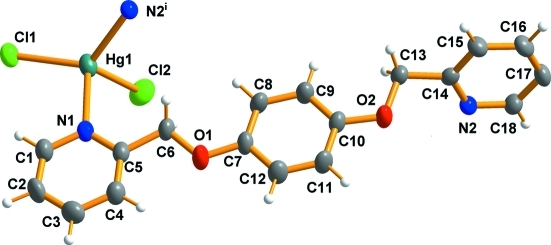
The asymmetric unit of the title compound. Displacement ellipsoids are drawn at the 50% probability level. [Symmetry code: (i) x, 1+y, z.]

**Fig. 2.**
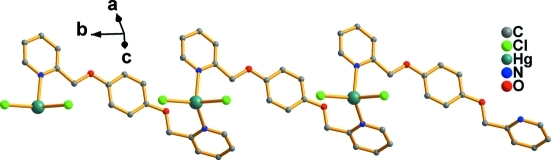
A view of the chain structure along the b axis. H atoms have been omitted for clarity.

## supplementary crystallographic information

### Comment

The metal-organic frameworks are determined by many factors, in which the organic ligands as building blocks and the kinds of metal ions are most important. Many pyridyl-containing ligands with strong coordination ability and functional characteristics have been studied over the recent years (Wang *et al.*, 2007). The flexible bipyridyl ligands can react with transition metals to construct helical-like structures. Recently, as a continuation of previous studies (Liu *et al.*, 2010*a*, *b*), we report here the crystal structure of the title compound.

In the title compound, the Hg^II^ atom is four-coordinated by two Cl atoms and two N atoms from two 1,4-bis(pyridin-2-ylmethoxy)benzene ligands, forming a tetrahedral geometry (Fig. 1). The Hg—Cl bond lengths are 2.398 (2) and 2.452 (2) Å, and Hg—N bond lengths are 2.282 (5) and 2.411 (5) Å. The angles around the Hg atom are in a range of 100.63 (13)–122.30 (12)°. The ligand shows a noncoplanar conformation, in which the dihedral angles between the two terminal pyridine rings and the linking benzene ring are 7.275 (17) and 74.020 (14)°. In the crystal, the flexible ligands link the Hg^II^ atoms into a chain running along [0 1 0], with an Hg···Hg separation of 10.335 (5)Å, which is equal to the *b* axis of the unit cell (Fig. 2). The chains are connected by C—H···O and C—H···Cl hydrogen bonds (Table 1).

### Experimental

1,4-Bis(pyridin-2-ylmethoxy)benzene was synthesized as the literature method (Liu *et al.*, 2010*a*,*b*). The title compound was produced by the reaction of ZnCl_2_ (0.50 mmol, 0.068 g) in water (5 ml) and 1,4-bis(pyridin-2-ylmethoxy)benzene (0.5 mmol, 0.146 g) in methanol (5 ml) under constant stirring. The mixture was filtered after stirring for about one hour. The filtate was maintained for about one week at room temperature to give colorless block-like crystals suitable for X-ray analysis.

### Refinement

The highest residual electron density was found at 0.87 Å from Hg1 atom and the deepest hole at 0.76 Å from Hg1 atom. H atoms were placed in calculated positions and treated as riding atoms, with C—H = 0.93 (aromatic) and 0.97 Å (methylene) and with *U*_iso_(H) = 1.2*U*_eq_(C).

### Crystal data

**Table:**

[HgCl_2_(C_18_H_16_N_2_O_2_)]	*Z* = 2
*M**_r_* = 563.82	*F*(000) = 536
Triclinic, *P*1	*D*_x_ = 2.027 Mg m^−^^3^
Hall symbol: -P 1	Mo *K*α radiation, λ = 0.71073 Å
*a* = 9.201 (5) Å	Cell parameters from 8238 reflections
*b* = 10.335 (5) Å	θ = 3.6–27.5°
*c* = 11.040 (6) Å	µ = 8.63 mm^−^^1^
α = 86.11 (2)°	*T* = 293 K
β = 66.51 (2)°	Block, colorless
γ = 73.860 (18)°	0.19 × 0.17 × 0.17 mm
*V* = 923.8 (9) Å^3^	

### Data collection

**Table:**

Rigaku R-AXIS RAPID diffractometer	4179 independent reflections
Radiation source: fine-focus sealed tube	3688 reflections with *I* > 2σ(*I*)
graphite	*R*_int_ = 0.051
ω scan	θ_max_ = 27.5°, θ_min_ = 3.6°
Absorption correction: multi-scan (*ABSCOR*; Higashi, 1995)	*h* = −10→11
*T*_min_ = 0.288, *T*_max_ = 0.325	*k* = −13→13
9080 measured reflections	*l* = −14→14

### Refinement

**Table:**

Refinement on *F*^2^	Primary atom site location: structure-invariant direct methods
Least-squares matrix: full	Secondary atom site location: difference Fourier map
*R*[*F*^2^ > 2σ(*F*^2^)] = 0.036	Hydrogen site location: inferred from neighbouring sites
*wR*(*F*^2^) = 0.122	H-atom parameters constrained
*S* = 1.16	*w* = 1/[σ^2^(*F*_o_^2^) + (0.0627*P*)^2^] where *P* = (*F*_o_^2^ + 2*F*_c_^2^)/3
4179 reflections	(Δ/σ)_max_ = 0.001
226 parameters	Δρ_max_ = 1.56 e Å^−^^3^
0 restraints	Δρ_min_ = −1.74 e Å^−^^3^

### Fractional atomic coordinates and isotropic or equivalent isotropic displacement parameters (Å^2^)

**Table:**

	*x*	*y*	*z*	*U*_iso_*/*U*_eq_	
C1	0.3961 (8)	1.3384 (6)	0.0220 (7)	0.0444 (16)	
H1	0.3896	1.4124	0.0692	0.053*	
C2	0.5080 (10)	1.3141 (7)	−0.1062 (7)	0.0503 (18)	
H2	0.5756	1.3705	−0.1448	0.060*	
C3	0.5187 (9)	1.2054 (7)	−0.1767 (7)	0.0505 (18)	
H3	0.5939	1.1867	−0.2640	0.061*	
C4	0.4164 (9)	1.1238 (7)	−0.1164 (7)	0.0438 (15)	
H4	0.4213	1.0497	−0.1626	0.053*	
C5	0.3057 (8)	1.1542 (6)	0.0148 (6)	0.0343 (12)	
C6	0.1907 (9)	1.0703 (6)	0.0877 (7)	0.0414 (15)	
H6A	0.2040	1.0418	0.1691	0.050*	
H6B	0.0774	1.1229	0.1100	0.050*	
C7	0.1385 (9)	0.8632 (6)	0.0600 (6)	0.0370 (14)	
C8	0.0193 (9)	0.8735 (6)	0.1867 (7)	0.0468 (18)	
H8	−0.0110	0.9494	0.2412	0.056*	
C9	−0.0548 (9)	0.7707 (6)	0.2322 (7)	0.0444 (16)	
H9	−0.1340	0.7771	0.3181	0.053*	
C10	−0.0126 (8)	0.6587 (5)	0.1515 (6)	0.0321 (12)	
C11	0.1058 (9)	0.6489 (6)	0.0227 (6)	0.0407 (15)	
H11	0.1345	0.5739	−0.0326	0.049*	
C12	0.1794 (9)	0.7515 (7)	−0.0215 (7)	0.0446 (16)	
H12	0.2580	0.7457	−0.1076	0.053*	
C13	−0.1985 (8)	0.5549 (6)	0.3153 (6)	0.0374 (13)	
H13A	−0.2948	0.6272	0.3208	0.045*	
H13B	−0.1615	0.5746	0.3811	0.045*	
C14	−0.2419 (7)	0.4243 (6)	0.3415 (6)	0.0324 (12)	
C15	−0.3957 (8)	0.4143 (7)	0.3561 (7)	0.0401 (14)	
H15	−0.4710	0.4882	0.3417	0.048*	
C16	−0.4359 (8)	0.2947 (7)	0.3918 (7)	0.0415 (15)	
H16	−0.5389	0.2874	0.4030	0.050*	
C17	−0.3210 (8)	0.1850 (6)	0.4111 (7)	0.0413 (15)	
H17	−0.3447	0.1027	0.4339	0.050*	
C18	−0.1700 (8)	0.2013 (6)	0.3954 (7)	0.0396 (14)	
H18	−0.0937	0.1290	0.4106	0.048*	
Cl1	0.2047 (2)	1.51507 (16)	0.35012 (17)	0.0436 (4)	
Cl2	0.2358 (3)	1.10739 (19)	0.4104 (2)	0.0581 (5)	
Hg1	0.13517 (3)	1.31748 (2)	0.31567 (2)	0.04162 (12)	
N1	0.2955 (6)	1.2612 (5)	0.0831 (5)	0.0361 (11)	
N2	−0.1291 (6)	0.3179 (5)	0.3590 (5)	0.0328 (11)	
O1	0.2274 (7)	0.9563 (5)	0.0059 (5)	0.0490 (13)	
O2	−0.0724 (6)	0.5467 (4)	0.1880 (5)	0.0450 (12)	

### Atomic displacement parameters (Å^2^)

**Table:**

	*U*^11^	*U*^22^	*U*^33^	*U*^12^	*U*^13^	*U*^23^
C1	0.046 (4)	0.033 (3)	0.047 (4)	−0.016 (3)	−0.007 (3)	−0.005 (3)
C2	0.056 (4)	0.044 (3)	0.045 (4)	−0.027 (3)	−0.006 (3)	0.006 (3)
C3	0.048 (4)	0.051 (4)	0.042 (4)	−0.016 (3)	−0.007 (3)	0.006 (3)
C4	0.055 (4)	0.041 (3)	0.044 (4)	−0.023 (3)	−0.022 (3)	0.005 (3)
C5	0.039 (3)	0.028 (3)	0.043 (3)	−0.014 (2)	−0.022 (3)	0.008 (2)
C6	0.055 (4)	0.035 (3)	0.044 (3)	−0.025 (3)	−0.021 (3)	0.005 (3)
C7	0.052 (4)	0.029 (3)	0.035 (3)	−0.018 (3)	−0.019 (3)	0.004 (2)
C8	0.052 (4)	0.029 (3)	0.040 (3)	−0.011 (3)	0.004 (3)	−0.015 (3)
C9	0.049 (4)	0.036 (3)	0.041 (3)	−0.019 (3)	−0.006 (3)	−0.003 (3)
C10	0.041 (3)	0.026 (2)	0.031 (3)	−0.009 (2)	−0.016 (2)	−0.005 (2)
C11	0.059 (4)	0.034 (3)	0.029 (3)	−0.018 (3)	−0.012 (3)	−0.009 (2)
C12	0.061 (4)	0.049 (4)	0.031 (3)	−0.028 (3)	−0.017 (3)	0.005 (3)
C13	0.043 (3)	0.030 (3)	0.039 (3)	−0.013 (3)	−0.014 (3)	0.006 (2)
C14	0.037 (3)	0.030 (3)	0.025 (3)	−0.006 (2)	−0.008 (2)	−0.002 (2)
C15	0.034 (3)	0.046 (3)	0.047 (4)	−0.018 (3)	−0.020 (3)	0.011 (3)
C16	0.036 (3)	0.044 (3)	0.044 (3)	−0.017 (3)	−0.010 (3)	−0.004 (3)
C17	0.041 (3)	0.040 (3)	0.044 (3)	−0.022 (3)	−0.011 (3)	0.005 (3)
C18	0.040 (3)	0.029 (3)	0.043 (3)	−0.008 (3)	−0.011 (3)	0.003 (3)
Cl1	0.0489 (8)	0.0469 (8)	0.0472 (9)	−0.0256 (7)	−0.0236 (7)	0.0053 (7)
Cl2	0.0738 (12)	0.0447 (9)	0.0561 (11)	−0.0065 (9)	−0.0337 (10)	0.0111 (8)
Hg1	0.04200 (17)	0.04003 (17)	0.05152 (19)	−0.01992 (12)	−0.02262 (13)	0.00908 (12)
N1	0.039 (3)	0.028 (2)	0.038 (3)	−0.011 (2)	−0.011 (2)	0.002 (2)
N2	0.035 (2)	0.030 (2)	0.035 (3)	−0.012 (2)	−0.014 (2)	0.001 (2)
O1	0.070 (3)	0.042 (2)	0.038 (2)	−0.034 (2)	−0.012 (2)	0.001 (2)
O2	0.063 (3)	0.040 (2)	0.038 (2)	−0.031 (2)	−0.015 (2)	0.0025 (19)

### Geometric parameters (Å, °)

**Table:**

C1—N1	1.338 (8)	C10—C11	1.393 (8)
C1—C2	1.367 (9)	C11—C12	1.374 (9)
C1—H1	0.9300	C11—H11	0.9300
C2—C3	1.369 (10)	C12—H12	0.9300
C2—H2	0.9300	C13—O2	1.410 (8)
C3—C4	1.380 (9)	C13—C14	1.491 (8)
C3—H3	0.9300	C13—H13A	0.9700
C4—C5	1.393 (9)	C13—H13B	0.9700
C4—H4	0.9300	C14—N2	1.349 (8)
C5—N1	1.341 (8)	C14—C15	1.391 (9)
C5—C6	1.502 (8)	C15—C16	1.375 (9)
C6—O1	1.413 (7)	C15—H15	0.9300
C6—H6A	0.9700	C16—C17	1.389 (10)
C6—H6B	0.9700	C16—H16	0.9300
C7—C8	1.380 (9)	C17—C18	1.387 (9)
C7—C12	1.383 (9)	C17—H17	0.9300
C7—O1	1.386 (7)	C18—N2	1.349 (8)
C8—C9	1.379 (9)	C18—H18	0.9300
C8—H8	0.9300	Hg1—Cl1	2.3981 (18)
C9—C10	1.378 (8)	Hg1—Cl2	2.452 (2)
C9—H9	0.9300	Hg1—N2^i^	2.282 (5)
C10—O2	1.385 (7)	Hg1—N1	2.411 (5)
			
N1—C1—C2	123.4 (6)	C11—C12—H12	119.5
N1—C1—H1	118.3	C7—C12—H12	119.5
C2—C1—H1	118.3	O2—C13—C14	109.4 (5)
C1—C2—C3	118.8 (6)	O2—C13—H13A	109.8
C1—C2—H2	120.6	C14—C13—H13A	109.8
C3—C2—H2	120.6	O2—C13—H13B	109.8
C2—C3—C4	119.1 (6)	C14—C13—H13B	109.8
C2—C3—H3	120.4	H13A—C13—H13B	108.3
C4—C3—H3	120.4	N2—C14—C15	121.4 (6)
C3—C4—C5	119.0 (6)	N2—C14—C13	116.8 (6)
C3—C4—H4	120.5	C15—C14—C13	121.6 (6)
C5—C4—H4	120.5	C16—C15—C14	119.7 (6)
N1—C5—C4	121.6 (6)	C16—C15—H15	120.1
N1—C5—C6	116.1 (5)	C14—C15—H15	120.1
C4—C5—C6	122.3 (6)	C15—C16—C17	119.3 (6)
O1—C6—C5	108.7 (5)	C15—C16—H16	120.3
O1—C6—H6A	109.9	C17—C16—H16	120.3
C5—C6—H6A	109.9	C18—C17—C16	118.2 (6)
O1—C6—H6B	109.9	C18—C17—H17	120.9
C5—C6—H6B	109.9	C16—C17—H17	120.9
H6A—C6—H6B	108.3	N2—C18—C17	122.7 (6)
C8—C7—C12	119.7 (6)	N2—C18—H18	118.6
C8—C7—O1	124.9 (5)	C17—C18—H18	118.6
C12—C7—O1	115.4 (6)	N2^i^—Hg1—Cl1	122.30 (12)
C9—C8—C7	119.8 (5)	N2^i^—Hg1—N1	107.69 (19)
C9—C8—H8	120.1	Cl1—Hg1—N1	102.88 (13)
C7—C8—H8	120.1	N2^i^—Hg1—Cl2	102.39 (14)
C10—C9—C8	120.6 (6)	Cl1—Hg1—Cl2	118.53 (8)
C10—C9—H9	119.7	N1—Hg1—Cl2	100.63 (13)
C8—C9—H9	119.7	C1—N1—C5	118.0 (5)
C9—C10—O2	125.7 (5)	C1—N1—Hg1	114.8 (4)
C9—C10—C11	119.9 (5)	C5—N1—Hg1	126.7 (4)
O2—C10—C11	114.3 (5)	C14—N2—C18	118.6 (5)
C12—C11—C10	119.1 (5)	C14—N2—Hg1^ii^	123.9 (4)
C12—C11—H11	120.4	C18—N2—Hg1^ii^	117.3 (4)
C10—C11—H11	120.4	C7—O1—C6	116.8 (5)
C11—C12—C7	121.0 (6)	C10—O2—C13	116.3 (4)

Symmetry codes: (i) *x*, *y*+1, *z*; (ii) *x*, *y*−1, *z*.

### Hydrogen-bond geometry (Å, °)

**Table:**

*D*—H···*A*	*D*—H	H···*A*	*D*···*A*	*D*—H···*A*
C2—H2···Cl1^iii^	0.93	2.82	3.692 (8)	157
C11—H11···O2^iv^	0.93	2.51	3.338 (9)	149

Symmetry codes: (iii) −*x*+1, −*y*+3, −*z*; (iv) −*x*, −*y*+1, −*z*.
